# Bioconversion of food wastewater into *Penicillium roqueforti* AF26 biomass proteins suitable for aquafeed

**DOI:** 10.3389/fmicb.2026.1857110

**Published:** 2026-07-29

**Authors:** Alejandro Ezequiel Figueroa, Luciana Melisa Del Gobbo, Agustina María Guerrero, María Beatriz Juarez, Victor Daniel Echegorry, Liliana Beatriz Villegas, Verónica Leticia Colin

**Affiliations:** 1 Planta Piloto de Procesos Industriales Microbiológicos (PROIMI-CONICET), San Miguel de Tucumán, Argentina; 2 Sección Química, Estación Experimental Agroindustrial Obispo Colombres (EEAOC), Las Talitas, Argentina; 3 Instituto de Química San Luis (INQUISAL-CONICET), Ciudad de San Luis, Argentina; 4 Facultad de Química, Bioquímica y Farmacia, Universidad Nacional de San Luis, Ciudad de San Luis, Argentina

**Keywords:** circular economy, food wastewater, *Penicillium roqueforti*, protein productivity, proximate analysis, sustainable aquaculture feed

## Abstract

The increasing need for aquaculture activities requires access to enough high-quality feed. Many commercial aquafeeds contain fishmeal as a main component, but this is a costly protein source. Therefore, shifting from traditional ingredients to alternative protein sources such as filamentous fungi biomass may alleviate concerns related to the cost of aquafeed. The aim of this study is to evaluate the biomass protein production by a filamentous fungus belonging to the genus *Penicillium* through the fermentation of food wastewater, specifically citrus wastewater and yogurt whey. In addition, the effect of supplementing wastewater with a nitrogen source (ammonium sulfate, (NH₄)₂SO₄, or urea, CO(NH₂)₂) on protein production performance was assessed. The fungus strain, named *Penicillium roqueforti* AF26, showed growth dependent on the increase in wastewater concentration, exhibiting greater biomass production on undiluted substrates: 3.83 ± 0.08 g/L on citrus wastewater and 22.7 ± 2.0 g/L on yogurt whey. Protein productivity enhanced significantly when wastewater was supplemented with (NH₄)₂SO₄, reaching values of 16.9 mg/L × h in citrus wastewater and 80.7 mg/L × h in yogurt whey. Proximate analysis of biomass with the highest protein productivity (biomass produced on undiluted yogurt whey supplemented with 1 g/L (NH₄)₂SO₄) revealed a total protein content (25.5%), fat (2.7%), crude fiber (3.6%), ash (15.0%), moisture (9.9%), and carbohydrate (46.9%) within reference ranges reported for commercial aquafeed. This study recorded the feasibility of a *P. roqueforti* strain to transform food wastewater into a sustainable and alternative protein source to be introduced in the aquaculture industry.

## 1 Introduction

The increased agricultural production to support food demands has raised major environmental concerns about deforestation, land degradation, and greenhouse gas emissions (Viana et al., 2022). In this context, understanding the current state of aquaculture and its potential is becoming a topic of academic research and public policy discussion. The Food and Agriculture Organization of the United Nations (FAO) has highlighted aquaculture as the fastest-growing major food production sector in the world (Food and Agriculture Organization of the United Nations, 2022), with fish serving as an important source of high-quality protein for humans. Nowadays, aquaculture supplies about half of the fish consumed globally (Rodríguez-Rodríguez et al., 2024), and it is expected to continue expanding to keep growing to satisfy increasing food demand (OECD/FAO, 2023). Unlike mammals, which mostly rely on carbohydrates as their main energy source, fish preferentially use protein (Konnert et al., 2022). Fishmeal has been the traditional ingredient in aquafeed because it provides protein, good palatability, and is easy to digest (Tignani et al., 2024). However, using fishmeal is not a long-term sustainable solution because it leads to overfishing, and the cost of obtaining it has been increasing (Fantatto et al., 2024). Consequently, the feed industry is crucial in aquaculture because feed is the largest input cost, comprising 40–60% of total production expenses in intensive farming systems (Engle et al., 2020).

Research has focused on plant- and insect-based protein alternatives (Wang Y. et al., 2023; Akter et al., 2024; Rimoldi et al., 2024; Rodríguez-Rodríguez et al., 2024; Fantatto et al., 2024), with microbial proteins being a promising but less explored option to replace fishmeal in aquaculture. Particularly, microbial proteins from sources like fungi can represent a significant fraction of dried biomass (Kamiri et al., 2023), making fungal biomass a nutritionally robust alternative protein source. Fungal biomass proteins can be produced more quickly and with fewer resources than plant and insect proteins, and they can be grown on waste materials through a biorefinery approach (Sar et al., 2024). This aligns with the European Union’s circular economy goals for managing wastewater, which aim to extract valuable products and reduce pollutants (Agovino et al., 2024). Citrus wastewater and yogurt whey (from yogurt processing) are liquid byproducts from the food industry, considered potentially harmful for water bodies and soil if improperly disposed of, as they have high organic content, low pH, and high suspended solids and conductivity (Lucia et al., 2025; Mirzakulova et al., 2025). The perspective of considering citrus wastewater and yogurt whey as waste byproducts has been changing as the intrinsic value of their components has been recognized and numerous possibilities for technological exploitation have emerged, such as essential oils, pectin, and flavonoids from citrus wastewater (Dikmetas et al., 2024), and production of lactic acid, biopolymers, bioethanol, or enzymes from whey (Delgado-Macuil et al., 2025). However, limited emphasis has been placed on the production of fungal biomass proteins from these wastewaters.

*Penicillium roqueforti* is a filamentous fungus best known for its role in blue cheese production (Metin, 2018, 2023), but it is also a prolific producer of diverse secondary metabolites with potential implications for food safety, biotechnology, and even medicine (Metin, 2023). The mycelial structure of *P. roqueforti*, like other fungi, includes proteins whose amounts are influenced by various factors, such as the substrate composition and the culture conditions (Maseko et al., 2025). Regarding substrate composition, the nitrogen content is crucial for protein synthesis, as it is an essential component in amino acids (Rovira-Alsina et al., 2025). Therefore, having sufficient nitrogen in the substrate directly aids in the formation of biomass proteins and other macromolecules that are necessary for the growth and metabolic activities of the fungus (Junaid et al., 2020). The aim of this study is to produce biomass protein potentially useful in aquaculture by fermentation of food wastewater, specifically citrus wastewater and yogurt whey, with a *P. roqueforti* strain. The effect of supplementing wastewater with two nitrogen sources, ammonium sulfate ((NH₄)₂SO₄) or urea (CO(NH₂)₂), on protein production was also evaluated.

## 2 Materials and methods

### 2.1 Sampling and fungus isolation

A batch of 60-day-ripening blue cheese, made using traditional methods, was microbiologically sampled. A portion of the inside of the cheese (5 g) was thoroughly mixed with 45 mL of distilled water to prepare a homogeneous cheese suspension. The suspension was then serially diluted from 1 × 10^−1^ to 1 × 10^−6^ concentrations. Subsequently, an aliquot (50 μL) of each diluted suspension was plated on PDA (potato dextrose agar) medium, incubating the plates at 25 ± 2 °C. At day 7 of incubation, the individual greenish colonies were selected and were re-sowed on new dishes with PDA, incubating for 7 days more to obtain a pure culture.

### 2.2 Morphological characterization

The macro- and microscopic morphological characterization of the pure culture, named AF26, was carried out in PDA medium on day 7. The macroscopic characteristics recorded were diameter, color, and surface of colonies (granular, like flour, mounting, slippery), texture, reverse color, exudate drops, etc. Microscopic examination of mycelium, after staining with methylene blue, was carried out with a light microscope (Nikon ECLIPSE 80i microscope, 100 × 1.3 NA objective), analyzing the presence of conidiophores, conidia shape, color, and arrangement.

### 2.3 Molecular characterization

The characterization of the isolate AF26 was carried out through PCR amplification and sequencing of the genomic regions of the *β*-tubulin gene. This gene is a highly standardized marker in the field of *Penicillium* taxonomy (Torres-García et al., 2022; Swant et al., 2023). It is capable of distinguishing between closely related species of the genus *Penicillium* while preserving conserved regions that facilitate reliable amplification. It produces stable and reproducible phylogenies without issues of pseudogenes or paralogs. The *β*-tubulin is one of the most robust loci for accurate species-level identification due to its widespread availability in reference databases.

For DNA extraction, the commercial “PURO Fungi” kit (PB-L Productos Bio-Lógicos) was used. Total genomic DNA was obtained from fungal mycelium growth during 48 h on YPD liquid culture. Cells were disrupted using a physical lysis method, grinding the frozen material with liquid nitrogen in a mortar until a fine powder is achieved. The primers for the β-tubulin gene were used: Bt2a (GGTAACCAAATCGGTGCTGCTTTC) and Bt2b (ACCCTCAGTGTAGTGACCCTTGGC) (Visagie et al., 2024). Polymerase chain reactions (PCR) were performed in a final volume of 20 μL containing 10–20 ng of DNA, 20 mM Tris–HCl buffer (pH 8.4), 0.2 mM each of dATP, dGTP, dCTP, and dTTP; 1.5 mM MgCl₂; 0.2 mM of each primer; and 1.5 U of Taq DNA polymerase. After an initial denaturation step at 95 °C for 5 min, the amplification cycles were carried out, and the resulting PCR products were analyzed using electrophoresis on a 1% (w/v) agarose gel. The final DNA concentration of PCR products was determined using an Epoch microplate spectrophotometer (BioTek), and sample integrity was assessed by 1% agarose gel electrophoresis, loading 5 μL of each sample. Amplicons were sequenced by the CERELA service and edited with Molecular Evolutionary Genetics Analysis (MEGA v7.0). The sequences were analyzed with BLASTn against NCBI databases (www.ncbi.nlm.nih.gov).

### 2.4 Source and characterization of food wastewater

Citrus wastewater used in this study was collected from a *local citrus processing industry* located in the department of Acheral, province of Tucumán, Argentina (26°45′00′′ S, 65°15′00′′ O), while yogurt whey was provided by a small family yogurt company located in the department of Lules, province of Tucumán, Argentina (26°55′05′′ S, 65°20′07′′ O). The physical and chemical analysis of food wastewater was conducted at Estación Experimental Agroindustrial Obispo Colombres (EEAOC) in Tucumán, Argentina. The testing followed standard procedures outlined in the Standard Methods for the Examination of Water and Wastewater (SMWW) and the Official Methods of Analysis from AOAC International (AOAC). The parameters analyzed included pH (SMWW 4500H + B) and electrical conductivity (EC) (SMWW 2510 B) at 25 °C. Total solids (TS) by drying at 105 °C (SMWW 2540 B) and total suspended solids (TSS) (SMWW 2540 D). Chemical oxygen demand (COD) (SMWW 5220 D) and biochemical oxygen demand (BOD) (SMWW 5210 B). Total nitrogen (NT) (SMWW 4500-N org.). Moisture (AOAC 934.01) and ash (AOAC 942.05). Refractive index (RI) (AOAC 921.08). Free fatty acid (AOAC 940.28) and density relative to water at 25 °C by calculation.

### 2.5 Biomass production

For biomass production, first the wastewater concentration (from 10 to 100%, v/v) and final spore concentration in the production medium (6 × 10^7^ CFU/mL or 6 × 10^7^ CFU/mL) were selected using a one-factor-at-a-time approach. The experiments were performed in 50 mL Erlenmeyer flasks at a final working volume of 10 mL. The production media were sterilized at 121 °C for 15 min, and then inoculated with fungal spores harvested from the PDA media. After incubating the flasks in an orbital shaker (150 rpm) for 96 h, the resulting biomass was collected by centrifugation (10,000 × *g* at 4 °C for 10 min). The collected biomass was repeatedly rinsed with distilled water and placed in an oven at 105 °C until it reached a constant weight. The amount of biomass produced was reported as the weight of the dried biomass in grams per liter of the culture.

Once wastewater concentration and spore concentration were selected, the effect of supplementing the food wastewater with a nitrogen source, (NH₄)₂SO₄ or CO(NH₂)₂, at a final concentration of 1.0 g/L was evaluated. The experiments were performed in 1000 mL Erlenmeyer shake flasks, in a final working volume of 200 mL. After the culture was incubated on an orbital shaker at 30 °C for 96 h, the biomass was collected by pouring the culture through a fine mesh sieve made of stainless steel with a pore area of 1 mm^2^. The biomass harvested was washed repeatedly with distilled water, was lyophilized, and was gravimetrically measured. The wastewater conversion efficiency was calculated and expressed as grams of fungal biomass per gram of initial COD.

### 2.6 Proximate analysis of biomass

The lyophilized biomass was analyzed by standard methods (Association of Official Analytical Chemists, 2012) to determine the following parameters: (a) total nitrogen content (Kjeldahl-Arnold-Gunning method), and then the total proteins (AOAC 920.12), for which the nitrogen content was multiplied by the conversion factor of 4.38 (Crisan and Sands, 1978). Protein productivity was also calculated and expressed as milligrams of protein produced per liter of culture per hour. (b) Total fat (or lipid) was determined by the Soxhlet gravimetric method extracting with petroleum ether (AOAC 945.39). (c) Crude fiber by digesting the sample with H₂SO₄ and NaOH for 30 min (AOAC 962.09). (d) Ash content by weight difference after calcining sample in a muffle at 550 °C for 4 h (AOAC 945.46). (e) Moisture by keeping the sample under reduced pressure at 100 °C for 24 h (AOAC 945.46). (f) Carbohydrates in indirect form through Equation 1:


$$\text{Total carbohydrates}=100–(\begin{array}{l} \text{Proteins}+\text{Total}\;\mathrm{Fat}\\ +\mathrm{Ash}+\text{Moisture} \end{array})$$ (1)

### 2.7 Statistical analysis

All statistical analyses were performed using Infostat (version 2020; InfoStat BibTex, Grupo InfoStat, FCA, Universidad Nacional de Córdoba, Córdoba, Argentina) software packages for Windows. Results were presented as mean ± standard deviation; all assays were performed in triplicate. Statistically significant differences among means were determined using one-way analysis of variance (ANOVA). Differences were considered significant at *p* < 0.05 with a 95% confidence interval. Subsequent comparisons among means were performed using Tukey’s post-hoc test. The main effects of the A (wastewater) and B (nitrogen source) factors and of their interactions on experimental results in terms of biomass production, total protein and protein productivity were identified using 2^2^ full factorial designs. The factors evaluated and their levels are presented in Supplementary materials S1, S2, with the “–” and “+” signs representing the low and high levels of each factor.

## 3 Results

### 3.1 Morphological and molecular characterization

**Figure 1:**
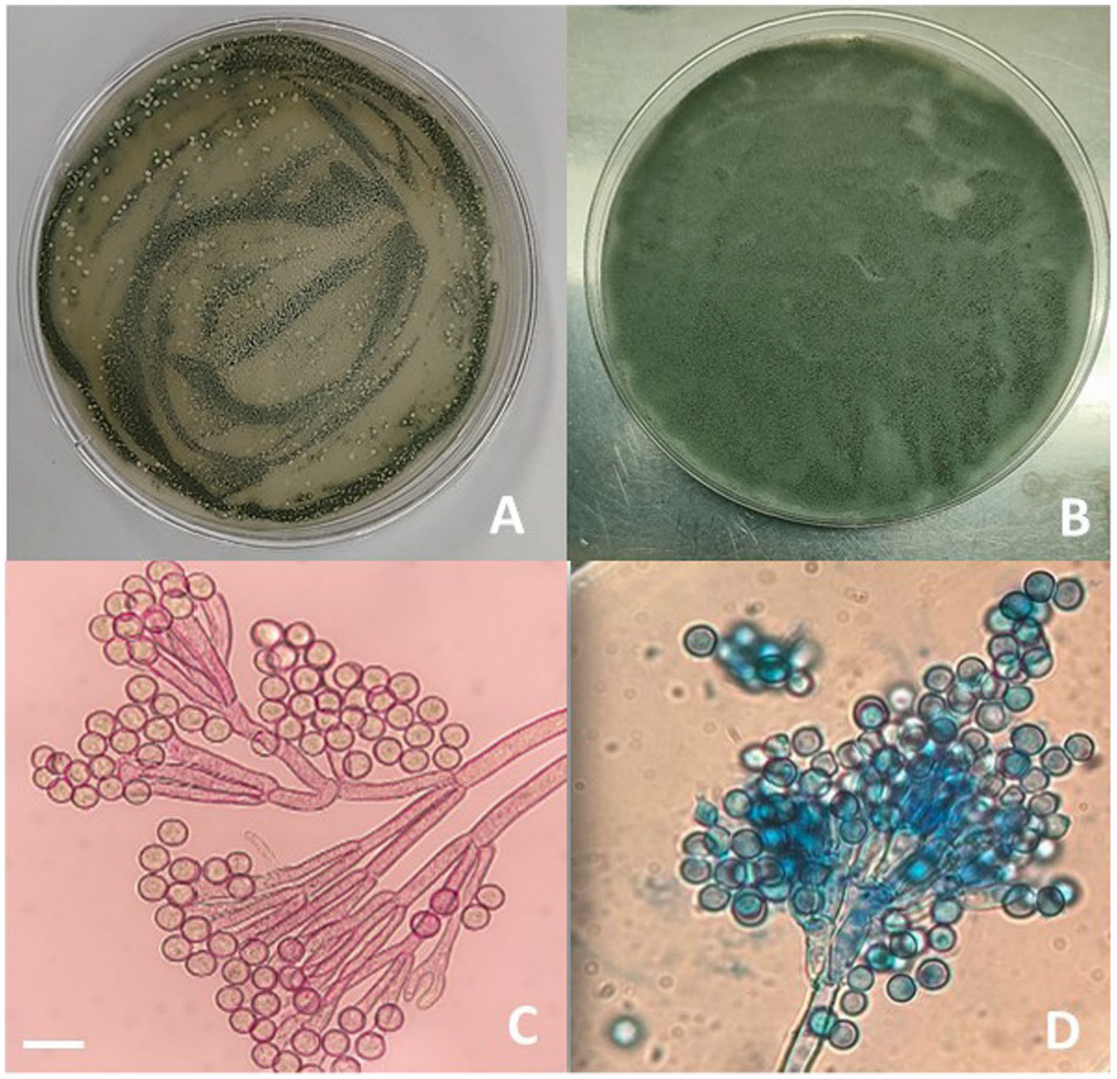
Isolates obtained from a cheese sample cultured on PDA medium **(A)**. The macroscopic characteristics of the isolate AF26 **(B)**. The microscopic characteristics of the isolate AF26, before **(C)** and after staining with methylene blue **(D)**. Microscopic observations will be carried out with a light microscope (Nikon ECLIPSE 80i microscope, 100 × 1.3 NA objective). **(C,D)** are at the same magnification, with the scale bar in **(C)** indicating 10 μm.

The isolates recovered from the sample cheese on PDA medium are depicted in Figure 1. Initial macroscopic examination allowed the differentiation of two isolate types (Figure 1A): white, mucous, yeast-like colonies, likely corresponding to yeasts commonly found in cheese environments, and dark green colonies, morphologically consistent with *Penicillium* spp. Dark green isolates subcultured on fresh PDA plates formed rapidly growing, low, velvety colonies (Figure 1B). Microscopic examination revealed characteristic brush-shaped conidiophores (penicilli) bearing chains of smooth, spherical conidia in basipetal succession (Figure 1C).

A partial *β*-tubulin gene sequence (421 bp) was obtained from the isolate and deposited in GenBank under accession number PZ260480 (Supplementary material S3). BLASTn analysis against the GenBank database identified *Penicillium roqueforti* as the closest taxon. The highest-scoring match corresponded to *P. roqueforti* strain CBS 479.84 (GenBank accession AY674382.1), with 94.50% nucleotide identity and 100% query coverage. In addition, all significant BLAST hits were assigned to *P. roqueforti*, with sequence identities of up to 97.3%, depending on query coverage. The molecular results, together with the characteristic colony morphology and microscopic features, consistently supported the identification of the isolate as *Penicillium roqueforti* AF26.

### 3.2 Effect of wastewater concentration and spore concentration on biomass production of *Penicillium roqueforti* AF26

The main physicochemical characteristics of citrus wastewater and yogurt whey used in this study to produce *P. roqueforti* AF26 biomass are shown in Tables 1, 2, respectively.

**Table 1:** Physicochemical characteristics of citrus wastewater.

Parameter	Value
pH	4.2
Electrical conductivity (EC), μS/cm	1,846
Total solid (TS), mg/L	6,566
Total suspended solids (TSS), mg/L	370
Chemical oxygen demand (COD), mg/L	18,285
Biochemical oxygen demand (BOD), mg/L	8,429
Total nitrogen (TN), mg/L	157
Moisture (%)	99.4
Ash (%)	0.101

**Table 2:** Physicochemical characteristics of yogurt whey.

Parameter	Value
pH	4.5
Chemical oxygen demand (COD), g/L	41.0
Biochemical oxygen demand (BOD), g/L	17.1
Total nitrogen (TN), mg/L	164
Refractive index (RI)	1.361
Density, g/cm^3^	1.077
Free fatty acid (FFAs), g KOH/100 g	0.83

The pH values were consistent with those reported for wastewater worldwide, presenting both an acidic nature (Table 1, 2). Citrus wastewater showed a high EC, which exceeded 1,500 μS/cm (Table 1). It also exhibited a high concentration of TS (Table 1), mainly due to the presence of organic matter precedent from fruit pulp, often leading to significant COD and BOD levels (Table 1). While the TN in citrus wastewater varies greatly, in this study, its value exceeded 100 mg/L (Table 1). Because fruits are mostly water, it was expected that their moisture content would be very high (more than 90%), while their ash content remains low (Table 1). Regarding yogurt whey, it presented a high COD level, primarily from lactose, proteins, and fats (Table 2). It also showed a notable content of TN, exceeding 150 mg/L (Table 2), which is attributed to the presence of whey proteins and non-protein nitrogen. The RI and density of the whey were found to be in line with values reported in other studies (de Wolf et al., 2024; Jessica et al., 2016), with values close to 1.3 for the RI and 1.000 g/cm^3^ for density (Table 2). In accordance with the literature, the level of free fatty acid in yogurt whey was found to be relatively low (Table 2), typically remaining under 1.0 g/100 g (Rocha-Mendoza et al., 2021).

**Figure 2:**
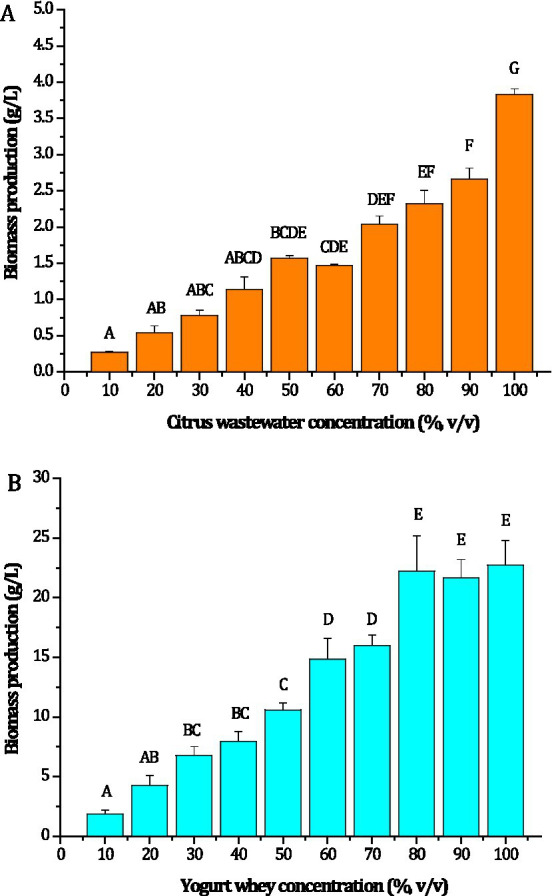
The effect of citrus wastewater **(A)** and yogurt whey **(B)** concentration on *Penicillium roqueforti* AF26 biomass production at 96 h of incubation. Results were analyzed using one-way analysis of variance (ANOVA). Bars with the same letter are not significantly different (*p* > 0.05, Tukey’s post-hoc test).

Once the food wastewater analysis had been carried out, these were used as cheap substrates for the production of fungal biomass proteins. As shown in Figure 2, *P. roqueforti* AF26 showed the ability to grow in all the concentrations tested for both types of wastewater. However, the magnitude of growth was significantly influenced (*p* < 0.05) by the type of wastewater and its concentration. In citrus wastewater, the maximum fungus growth was observed in the undiluted effluent, with a biomass production approaching 4 g/L (Figure 2A). The fungus growth in yogurt whey was substantially greater than in citrus wastewater, with the maximum biomass production (~22 g/L) being detected at concentrations that ranged between 80 and 100% (Figure 2B).

**Figure 3:**
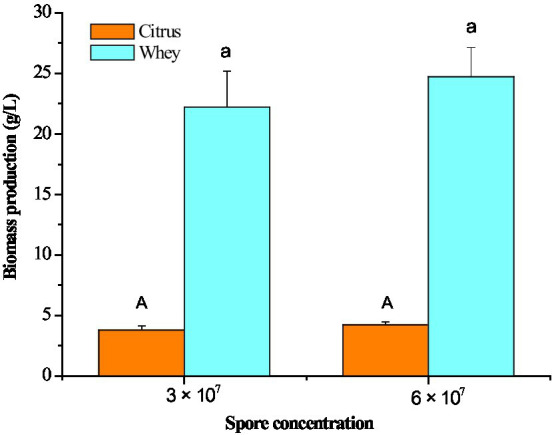
The effect of spore concentration on *Penicillium roqueforti* AF26 biomass production after 96 h of incubation in both citrus wastewater and yogurt whey. Results were analyzed using one-way analysis of variance (ANOVA). Bars with the same letter are not significantly different (*p* > 0.05, Tukey’s post-hoc test).

Regarding variations inherent to the microorganism, increasing the initial spore concentration in the wastewaters by double did not lead to a significant change in the amount of fungal biomass produced (*p* > 0.05) (Figure 3).

### 3.3 Factorial design using (NH₄)₂SO₄ as a nitrogen source

**Figure 4:**
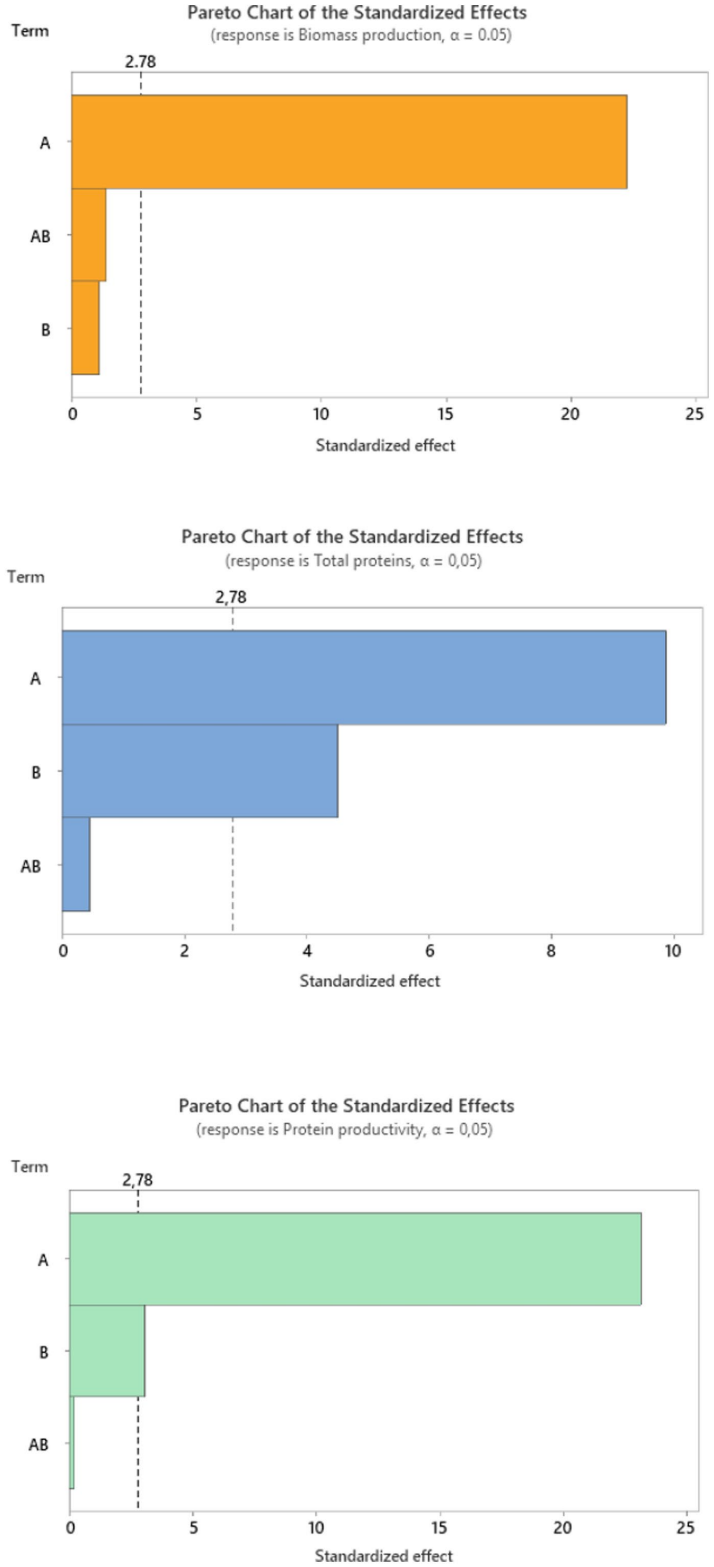
Pareto charts showing standardized effects of A (wastewater) and B (nitrogen source) factors for experimental results, using (NH_4_)_2_SO_4_ as a nitrogen source. The vertical lines represent a reference score, with the bars crossing this score considered statistically significant.

Table 3 shows the matrix for the first factorial design applied in this study to evaluate the effect of factor A (wastewater) and factor B (nitrogen source), added as (NH₄)₂SO₄, on biomass production, total proteins, and protein productivity of *P. roqueforti* AF26. Pareto charts from Figure 4 illustrated the standardized effects of factors A and B and their interactions on experimental results. The signs of the main effects of each factor individually and of the AB interaction effect are presented in Table 4.

**Table 3:** Matrix for the first full factorial design using (NH_4_)_2_SO_4_ as a nitrogen source, and experimental results measured at 96 h of incubation.

Run	Factor		Experimental results		
	A (wastewater)	B (nitrogen source)	Biomass production (g/L)	Total proteins (%)	Protein productivity (mg/L·h)
1	−	−	4.7 ± 0.1	14.5 ± 2.1	7.1 ± 0.8
2	−	+	6.5 ± 0.1	18.3 ± 0.1	12.4 ± 0.1
3	+	−	21.5 ± 1.4	22.4 ± 0.3	50.2 ± 4.0
4	+	+	21.3 ± 1.4	25.5 ± 0.3	56.4 ± 3.5

“–” represents “citrus wastewater or without (NH₄)₂SO₄ supplementation,” whereas “+” represents “yogurt whey or supplementation of 1 g/L (NH₄)₂SO₄”.

**Table 4:** Estimated effect analysis for experimental results, using (NH_4_)_2_SO_4_ as a nitrogen source.

Term	Biomass production (g/L)		Total protein (%)		Protein productivity (mg/L·h)	
	Effects	*T*-values (*p*-values)	Effects	*T*-values (*p*-values)	Effects	*T*-values (*p*-values)
Wastewater (A)	15.800	22.23 (<0.001)	7.550	9.87 (0.001)	43.550	23.15 (<0.001)
Nitrogen source (B)	0.800	1.13 (0.323)	3.450	4.51 (0.011)	5.750	3.06 (0.038)
AB	−1.000	−1.41 (0.232)	−0.350	−0.46 (0.671)	0.400	0.21 (0.842)

The *p*-values from Figure 4 and Table 4 indicate that biomass production was only significantly affected (*p* < 0.05) by factor A. Cultivating *P. roqueforti* AF26 in yogurt whey resulted in an average biomass production almost 5 times higher than that obtained in citrus wastewater, regardless of (NH₄)₂SO₄ supplementation (Table 3). The conversion efficiency, i.e., the grams of fungal biomass produced by grams of initial DQO, was 0.306 g/g for citrus wastewater and 0.522 g/g for yogurt whey. This implies an increase of 70.5% in *P. roqueforti* AF26 efficiency when grown in the lactic residue. The total proteins and protein productivity were significantly influenced (*p* < 0.05) by both factors (Figure 4 and Table 4). When factor A is considered, the average value of total proteins and protein productivity recorded in *P. roqueforti* AF26 growing on yogurt whey increased by 46 and 446%, respectively, compared to those obtained from citrus wastewater (Table 3). Regarding the influence of factor B, a significant increase (*p* < 0.05) in total proteins and protein productivity associated with (NH₄)₂SO₄ supplementation was observed, both in citrus wastewater and yogurt whey (Tables 3, 4 and Figure 4). Finally, AB interaction did not have statistically significant effects (*p* > 0.05) on the variables studied (Figure 4 and Table 4).

### 3.4 Factorial design using CO(NH₂)₂ as a nitrogen source

**Figure 5:**
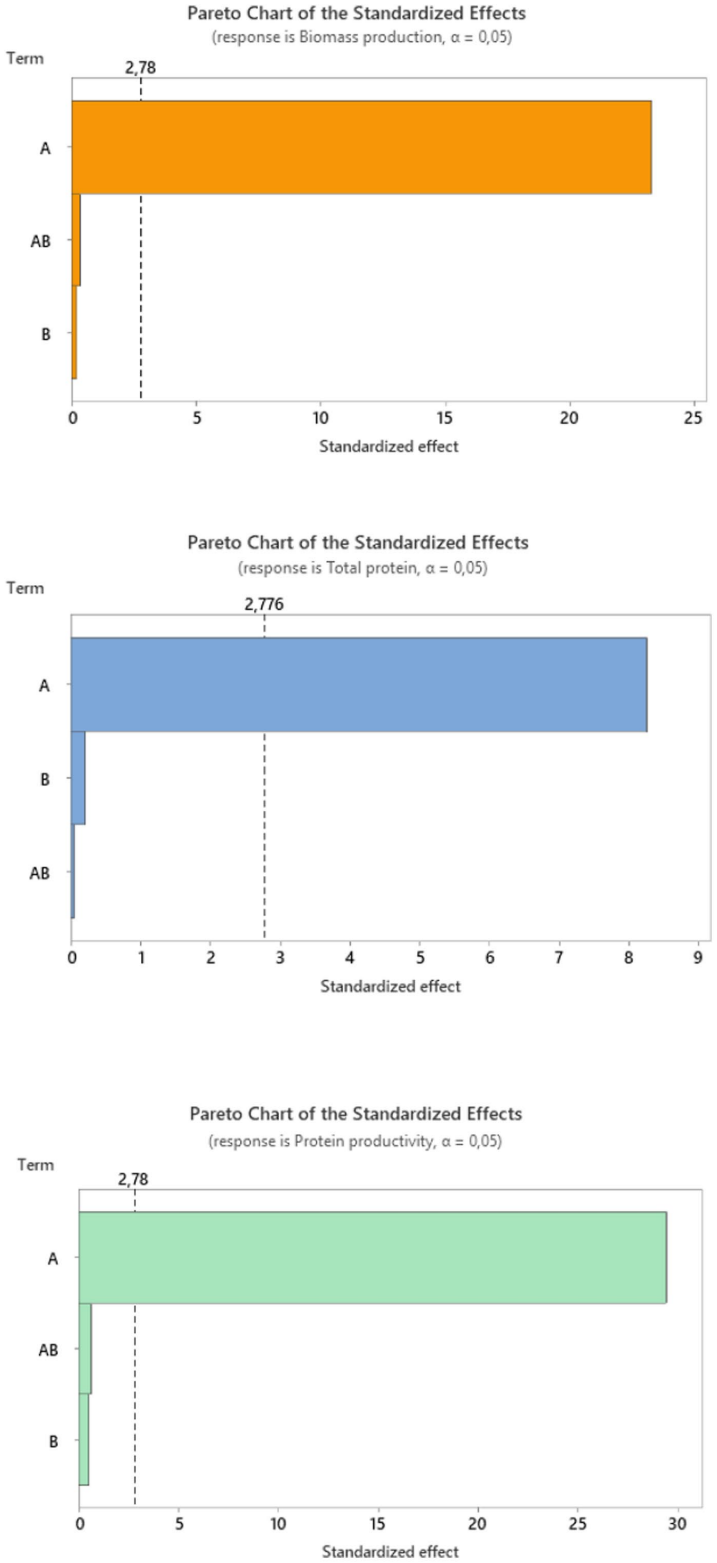
Pareto charts showing standardized effects of A (wastewater) and B (nitrogen source) factors for experimental results, using CO(NH_2_)_2_ as a nitrogen source. The vertical lines represent a reference score, with the bars crossing this score considered statistically significant.

Table 5 shows the matrix for the second factorial design carried out in this study to evaluate the effect of factor A and factor B (added as CO(NH₂)₂) on experimental results. Pareto charts from Figure 5 illustrate the standardized effects of both factors and their interactions on biomass production, total proteins, and protein productivity of *P. roqueforti* AF26. The signs of the main effects of each factor individually and of the AB interaction effect are presented in Table 6.

**Table 5:** Matrix for the second full factorial design using CO(NH_2_)_2_ as a nitrogen source, and experimental results measured at 96 h of incubation.

Run	Factor		Experimental results		
	A (wastewater)	B (nitrogen source)	Biomass production (g/L)	Total proteins (%)	Protein productivity (mg/L·h)
1	−	−	4.7 ± 0.1	14.5 ± 2.1	7.1 ± 0.8
2	−	+	4.8 ± 0.1	14.4 ± 0.1	7.2 ± 0.3
3	+	−	21.5 ± 1.4	22.4 ± 0.3	50.2 ± 4.0
4	+	+	21.1 ± 1.4	22.2 ± 1.6	48.6 ± 0.4

“–” represents “citrus wastewater or without CO(NH₂)₂ supplementation,” whereas “+” represents “yogurt whey or supplementation of 1 g/L CO(NH₂)₂”.

**Table 6:** Estimated effect analysis for experimental results, using CO(NH_2_)_2_ as a nitrogen source.

Term	Biomass production (g/L)		Total protein (%)		Protein productivity (mg/L·h)	
	Effects	*T*-values (*p*-values)	Effects	*T*-values (*p*-values)	Effects	*T*-values (*p*-values)
Wastewater (A)	16.550	23.29 (<0.001)	7.850	8.26 (0.001)	42.250	29.43 (<0.001)
Nitrogen source (B)	−0.150	−0.21 (0.843)	−0.200	−0.21 (0.844)	−0.750	−0.52 (0.629)
AB	−0.250	−0.35 (0.743)	−0.050	−0.05 (0.961)	−0.900	−0.63 (0.565)

The *p*-values from Figure 5 and Table 6 indicate that the three parameters evaluated were only significantly affected (*p* < 0.05) by factor A. Growing *P. roqueforti* AF26 on yogurt whey enhanced biomass production, total proteins, and protein productivity by 348, 54, and 590%, respectively, in comparison to the values obtained from citrus wastewater (Table 5). Regarding conversion efficiency, it was 0.260 grams per gram for citrus wastewater and 0.520 grams per gram for yogurt whey, which implies a 100% increase when lactic residue is used.

### 3.5 Proximate analysis of *Penicillium roqueforti* AF26 biomass produced from yogurt whey

**Figure 6:**
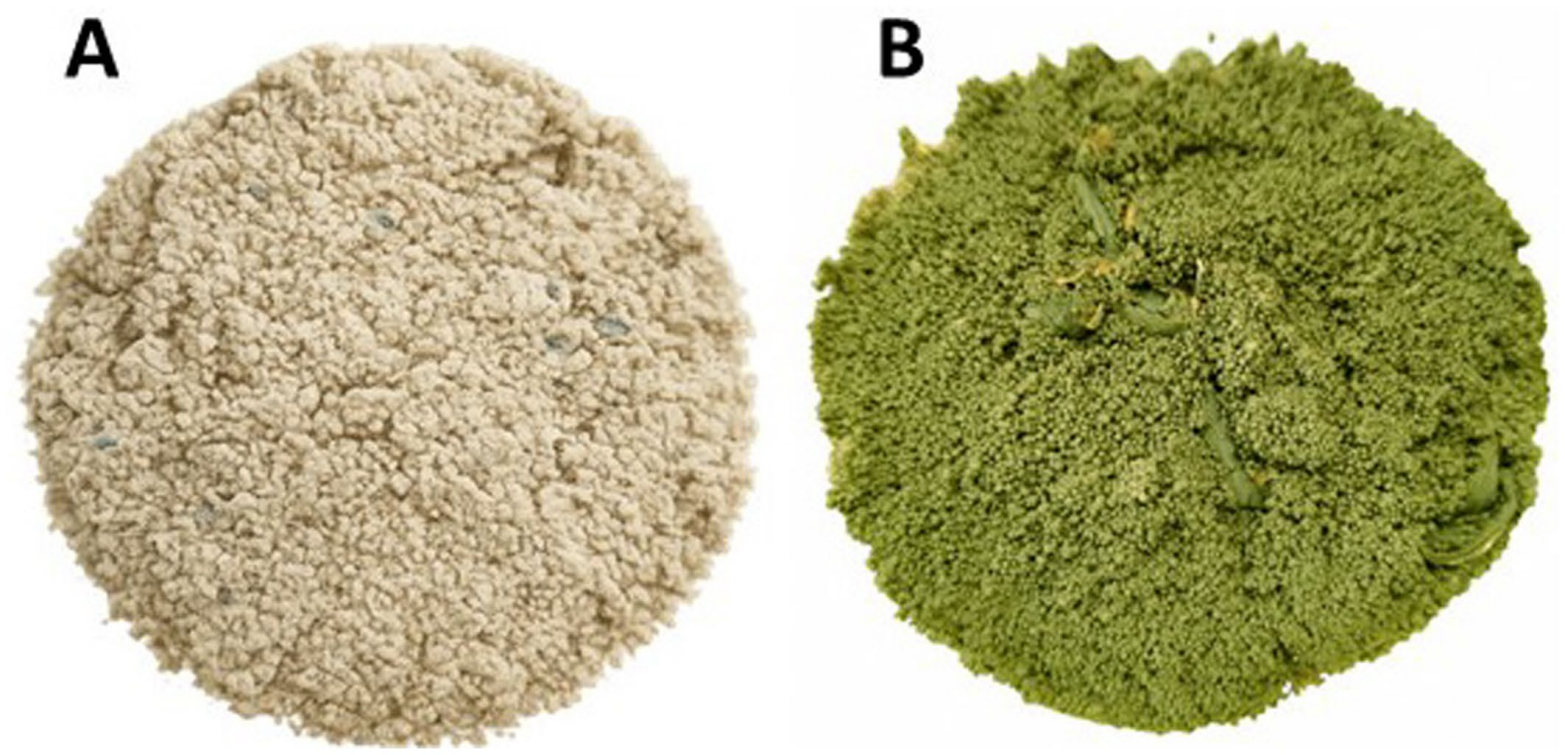
Lyophilized biomass of *Penicillium roqueforti* AF26 produced from citrus wastewater **(A)** and yogurt whey **(B)**.

Figure 6 shows the lyophilized biomasses of *P. roqueforti* AF26 produced from citrus wastewater (Figure 6A) and yogurt whey (Figure 6B). The factorial designs conducted in this study indicated that lactic residue is a more suitable substrate than citrus wastewater in terms of protein productivity (Tables 3–6). Therefore, the proximal analysis of yogurt whey-sourced biomasses labeled B_1_ (without nitrogen source supplementation, control), B_2_ (with supplementation of 1 g/L (NH₄)₂SO₄), and B_3_ (with supplementation of 1 g/L CO(NH₂)₂) was conducted to identify additional nutrients required in aquafeed beyond protein (Table 7). Determination of total fat content reveals the following order: B_2_ > B_1_ ~ B_3_. This indicates that adding 1 g/L of (NH₄)₂SO₄ resulted in a moderate increase in lipid levels, while supplementing 1 g/L of CO(NH₂)₂ caused a slight decrease compared to the control. The fiber content in B_1_, B_2_, and B_3_ does not show substantial variations associated with the nitrogen source added to the yogurt whey. However, the ash level in B_2_ and B_3_ increased more than 3-fold compared to B_1_. It is important to note that the moisture content remained below 10% in the three biomasses analyzed. Finally, the addition of nitrogen sources, particularly (NH₄)₂SO₄, led to a reduction in the total carbohydrate content of the biomass.

**Table 7:** Proximate composition of *Penicillium roqueforti* AF26 biomass produced from yogurt whey and nutrient reference ranges for commercial aquafeeds.

Parameter (%)	Biomass			Commercial aquafeed ^a,b,c,d^
	B_1_	B_2_	B_3_	
Total proteins	22.4 ± 0.3	25.5 ± 0.3	22.2 ± 1.6	25.0–40.0
Total fat	2.0 ± 0.0	2.7 ± 0.2	1.7 ± 0.1	1.0–10.0
Crude fiber	3.4 ± 0.8	3.6 ± 0.5	3.3 ± 0.3	<8.0
Ash	4.2 ± 0.2	15.0 ± 1.2	14.2 ± 1.0	<18.0
Moisture	9.2 ± 1.0	9.9 ± 0.4	7.4 ± 0.4	<14.0
Carbohydrates	62.2 ± 0.3	46.9 ± 0.3	54.5 ± 1.6	25.0–50.0

B_1_, Biomass produced without nitrogen source supplementation; B_2_, Biomass produced with supplementation of 1 g/L (NH₄)₂SO₄. B_3_, Biomass produced with supplementation of 1 g/L CO(NH₂)₂. ^a^Boyd (2018). ^b^Soong et al. (2016). ^c^Russo and Yanong (2013). ^d^Jobling (2012).

## 4 Discussion

### 4.1 Fungal biomass protein as a sustainable alternative for the aquaculture industry

In aquaculture, fish are the most significant organisms, making up about 50% of the total aquaculture output (Mair et al., 2023). Fish require a diet that includes protein as the main nutrient, which is essential for their growth, reproduction, and other normal bodily functions. It is essential to ensure that fish feed meets all required standards, as this directly influences the productivity of aquaculture species (Khan et al., 2025). However, the specific protein dietary needs of fish can change depending on the species, age, stage in their life cycle, sex, and the environment they are in (Sugiura, 2025).

Although fishmeal has traditionally been the main source of protein in fish feed, challenges in its production have caused the aquaculture industry to seek more affordable and sustainable alternative protein sources to replace fishmeal. Fungal mycelium proteins offer a promising possibility to serve as a replacement or supplement for fishmeal in aquafeed (Rulli et al., 2021; Kamiri et al., 2023; Del Gobbo et al., 2023; Onomu and Okuthe, 2024). It is noteworthy that the production of these proteins requires considerably less land and water compared to proteins obtained from animals and plants (Geerits et al., 2025). Fungal biomass proteins exist in various forms, including enzymes, storage proteins, cell wall proteins, and transporters (Wang B. et al., 2023), and their levels differ based on the type of fungus, as different species have unique cell structures (Karimi et al., 2018). Fungi from genera like *Aspergillus*, *Fusarium*, *Neurospora*, and *Rhizopus*, which are generally recognized as safe (GRAS), are considered potential sources of protein for animal feed, including feed used in aquaculture (Karimi et al., 2021). Species within the *Penicillium* genus, however, have not been extensively studied for such applications. As a result, there remain a significant number of research opportunities in this field.

Despite progress in this area, the primary limitation of using fungal biomass as a feed ingredient is linked to the production of mycotoxins. These secondary metabolites commonly produced by species in the genera *Aspergillus*, *Penicillium*, and *Fusariu*m are toxic substances that can reduce productivity in fish farming, leading to slower growth, increased disease susceptibility, and higher fish mortality (Puvača et al., 2024). It is known that *P. roqueforti* to produce secondary metabolites, some of which are classified as mycotoxins. Among these, the PR toxin is the most toxic and has been associated with damage to vital internal organs, gastrointestinal perturbations, carcinogenicity, immunotoxicity, necrosis, and enzyme inhibition (Dubey et al., 2017). However, it is chemically unstable and tends to degrade into a compound with reduced toxicity (Chang et al., 1993). *Penicillium roqueforti* also generates other mycotoxins such as roquefortine C and mycophenolic acid, but these compounds exhibit low cytotoxic properties compared to other mycotoxins that are regulated in food by the European Union, such as aflatoxins, ochratoxin A, and patulin (Commission Regulation European Union N° 2023/905). In this context, the use of *P. roqueforti* in aquafeed formulations is innovative and is supported by its well-documented safety in food and its accessibility in conventional industrial processes (Metin, 2023). Nevertheless, assessing the levels of mycotoxins is essential to ensure the production of nutritionally safe biomass for feeding purposes.

In addition to mycotoxins, another challenge of using fungal biomass as a feed ingredient in a real scenario is the high cost of cultivation media. This issue has led to efforts aimed at finding more affordable substrates, which is crucial for ensuring the long-term sustainability of large-scale production of fungal biomass protein. One approach in this direction involves developing biorefinery processes that focus on using organic-rich waste and industrial by-products as sources of nutrients for microbial growth (Rulli et al., 2021; Del Gobbo et al., 2023). This approach successfully tackles both the economic and environmental hurdles of large-scale production, generating affordable, nutrient-rich fungal biomass while mitigating waste disposal issues.

### 4.2 Biorefinery approach for production of fungal biomass protein

Every day, large quantities of organic-rich waste and industrial by-products are generated. These complex materials contain high levels of nutrients that can be recovered through a biological process known as bioconversion. Microorganisms, especially filamentous fungi, can decompose these complex materials because of their advanced enzyme systems (Giwa et al., 2023), and they use the nutrients obtained from this process to help them grow and develop. Additionally, the hyphal network of fungi can shield the delicate internal structures from the harmful effects of these complex substances, which also possess contaminating characteristics.

Using fungi to transform wastewaters into protein-rich biomass is becoming a cost-effective alternative to traditional protein production methods. This biotechnological approach, known as biorefinery, not only enhances the economic sustainability of production processes but also helps reduce environmental impact by decreasing the release of pollutants. Even though there are some challenges when using fungal biomass protein produced from wastewater as animal feed, like concerns about safety, regulatory compliance, and public acceptance, the benefits in terms of sustainability, nutrient recycling, and environmental protection are significant. Recent studies indicate that European countries have a favorable view of fungal protein-based foods and feeds, as they make use of resources that would otherwise be wasted (Hellwing, 2023).

Argentina ranks among the top 10 global producers of citrus fruits and is one of the four leading countries in lemon production. It is also recognized as the world’s first country to process lemons (Ferrero et al., 2022). In addition, our country is a leading producer of yogurt, playing a significant role in the dietary habits of Argentinians (Britos et al., 2024), as it appeals to consumers across various age groups. Citrus wastewater and yogurt whey, also known as acid whey, are considered significant sources of environmental pollution. This is primarily due to their high content of organic matter and their low pH levels. Many microorganisms are unable to grow in acidic conditions; therefore, it is necessary to adjust the initial pH of acidic wastewater before using it as a culture medium. However, filamentous fungi are either adapted to acidic conditions or can tolerate acidity (Thuy et al., 2025). Consequently, wastewaters with low pH levels, as used in this study, can be successfully utilized in fungal biorefinery.

Most industrial wastewater requires a previous dilution to promote optimal microbial growth, as it often contains substances that can be harmful to microorganisms (Nagi et al., 2020). The current study showed that increasing the concentration of food wastewater promoted the growth of *P. roqueforti* AF26. Based on this finding, we proceeded with undiluted food wastewater in further experiments, as it offers a practical benefit for potential industrial use by reducing water usage. The current work also revealed that increasing the concentration of *P. roqueforti* AF26 spores in the production medium did not have a significant impact on the amount of biomass generated. This result might be because the nutrients in the substrate are limited (Lou et al., 2024). It may also be related to the cell density phenomenon, known in mycology as self-inhibition of spore germination. In this process, spores stop themselves from germinating when they are found in high concentrations due to pre-existing self-inhibitors (Takeuchi et al., 2024).

Filamentous fungi are effective at absorbing nutrients like nitrogen from wastewater, and the presence of nitrogen typically has a direct impact on their growth and protein production. Toghiani et al. (2025) reported that supplementing a nitrogen source, sodium nitrate (NaNO₃), to the pistachio dehulling waste used as a growth substrate improved the biomass production and protein content of *Neurospora intermedia*. Similarly, Cao et al. (2025) observed that adding mainly organic nitrogen sources enhanced the growth of *Aspergillus awamori* when using almond hull extract as a culture medium. In the current work, it was found that supplementing (NH₄)₂SO₄ or CO(NH₂)₂ to citrus wastewater and yogurt whey did not result in a higher production of *P. roqueforti* AF26 biomass. However, adding (NH₄)₂SO₄ to food wastewater significantly increased the protein content of mycelium. This might happen because inorganic nitrogen sources, like NH^4+^, are simple molecules that require a relatively low energetic cost of assimilation and protein production by fungi compared to more complex organic nitrogen sources (Howard et al., 2024). This finding can be viewed as a benefit from an economic perspective, as inorganic nitrogen sources are generally less expensive than organic nitrogen sources due to their simpler composition and reduced production costs.

### 4.3 Components of proximate analysis and its value in aquaculture

In aquaculture, feed accounts for more than half of the total operating expenses, and among the dietary components, protein is the most costly element (Hughes et al., 2025). Since protein requirements vary by species and life stage (Zhang et al., 2025), commercial aquafeeds usually maintain a protein level between 25 and 45% (Boyd, 2018). Diets with 20–30% protein are typically used for herbivorous or omnivorous fish and later life stages, whereas juveniles require higher levels (30–50%) to support rapid growth [National Research Council (NRC), 2011]. The present work demonstrates that yogurt whey was a more effective substrate than citrus wastewater for *P. roqueforti* AF26 growth. This indicates that dairy by-products provide sufficient residual nutrients to support fungal metabolism. The three biomasses produced (B_1_, B_2_, and B_3_) exceeded 20 g/L, showing total protein levels reached 22–25% alongside an average protein productivity of 52 ± 4 mg/L·h. Consequently, *P. roqueforti* AF26 grown on yogurt whey could potentially serve as a maintenance diet for freshwater farmed fish, contributing to a more sustainable aquaculture.

Beyond protein, the quality of aquafeed also depends on other nutrients that must be considered when evaluating its overall nutritional value. Proximate analysis involves the determination of the basic chemical components present in a sample, which provide essential information about the nutritional composition of feed and feed ingredients used in aquaculture. By understanding these components, it becomes possible to evaluate the quality and appropriateness of feed for various fish species, which is crucial for supporting their best possible growth, health, and overall productivity within aquaculture systems. In other words, the value of proximate analysis lies in its ability to support informed decision-making regarding feed formulation and management practices in aquaculture.

Lipids are important macronutrients in fish feed, as they play key physiological roles and help make the most efficient use of dietary protein (Ning et al., 2023). Lipids act as a source of energy and supply necessary fatty acids, intact phospholipids, and cholesterol, all of which are important for normal growth, development, and health (Fei et al., 2024). Like proteins, the lipid requirement is very variable and depends mainly on the fish species. Lipid inclusion in fish feeds is usually provided through fish oil. However, excessive reliance on this resource causes sustainability problems linked to the overexploitation of small pelagic fishery stocks, being the most costly nutrient in aquaculture after protein (Monteiro et al., 2024). As a result, cheaper lipid sources like fungal biomass produced from industrial wastewaters could help improve the long-term sustainability of aquaculture. Filamentous fungi, such as *Aspergillus oryzae*, have been reported as moderate lipid accumulators, with lipid content typically ranging from 2 to 5% of dry weight (Karimi et al., 2019). Other fungi like *Neurospora intermedia* are regarded as good lipid producers, capable of accumulating total fat content exceeding 20% under certain culture conditions (Hoxha et al., 2026). The present work indicates that adding different nitrogen sources to yogurt whey has opposing effects on the lipid content of *P. roqueforti* AF26. The inclusion of (NH₄)₂SO₄ increased the total fat content in the fungus biomass (B_2_), bringing it within the recommended range for many farmed omnivorous fish (1–10%) (Soong et al., 2016). In contrast, the addition of CO(NH₂)₂ led to a 15% reduction in biomass lipid content (B_3_) compared to reference biomass (B_1_).

Dietary fiber, which consists of non-digestible components such as chitin and glucan, plays an important role in keeping the digestive system in good condition, helping the fish absorb nutrients effectively, and contributing to the overall well-being of the animal. Fibers also contribute positively to animal health due to their hypolipidemic properties. However, fiber is typically required in amounts less than 8% in most aquafeeds (Soong et al., 2016), since excessive amounts could reduce digestibility and nutrient availability, particularly for carnivorous fish (Bonvini et al., 2018). Fungal biomass can present high fiber content, reporting in some cases values higher than 30% of dry weight (Nordlund et al., 2024). This study demonstrates that the *P. roqueforti* AF26 biomass generated in yogurt whey has a similar fiber content regardless of the addition of nitrogen sources. It is important to note that in the three biomasses (B_1_, B_2_, and B_3_), the fiber content was less than 4%.

Ash in aquafeed refers to the total amount of inorganic minerals, primarily including calcium, phosphorus, magnesium, and potassium. This fraction typically constitutes between 8 and 12% of the feed’s dry matter, but it may be higher when lower-quality ingredients are used (Camperio et al., 2025). Although these minerals are important for maintaining bone health and supporting metabolic processes, an excess of ash can lower the energy content of the feed, making nutrients less available for proper growth (Eggink et al., 2024). Our study indicated that adding nitrogen sources such as (NH₄)₂SO₄ or CO(NH₂)₂ to yogurt whey leads to an increase in ash content in the *P. roqueforti* AF26 biomass. Thus, the 18% limit set as the highest level allowed for commercial aquafeed was not broken in any of the biomass samples (B_1_, B_2_, and B_3_).

Moisture content is a key factor, as it has a great impact on the stability and preservation of aquafeed. When the moisture level exceeds 14%, it can greatly increase the risk of production of mycotoxin by toxigenic fungi (Russo and Yanong, 2013). Considering that fungal biomass typically contains a high amount of water, removing moisture from mycelium through dewatering is a key step, as it helps to minimize the risk of deterioration. In this regard, we found that lyophilization process of B_1_, B_2_, and B_3_ reduces the moisture content of the biomass to below 10%.

Carbohydrates are also essential macronutrients in feed for aquaculture and constitute the most economic energy source, being vital for the protein-sparing effect (Fei et al., 2024). In comparison with lipids, most species of fish use them less efficiently. Carnivorous species, in particular, have a limited capacity to digest carbohydrates and require less than 20% inclusion in their diet. This is because their digestive systems are less well-suited to process them than those of herbivorous and omnivorous species (Ning et al., 2023). The present investigation determined that the total carbohydrate content in B_2_ was approximately 47% of its dry weight. This level is within the acceptable range for cultivated freshwater species, which could tolerate up to 50% of this macronutrient (Jobling, 2012).

## 5 Conclusion

This study shows that *P. roqueforti* AF26 can effectively bioconvert citrus wastewater and, to a greater extent, yogurt whey into fungal biomass proteins. The strain exhibited robust growth across all ranges of food wastewater concentrations, with undiluted yogurt whey supporting the highest biomass and protein productivities. Nitrogen supplementation, added as (NH₄)₂SO₄, further enhanced biomass protein productivity, highlighting the importance of nutrient balance in optimizing fungal bioconversion processes. Proximate analysis confirmed that the resulting biomass possesses a nutritional profile suitable for aquafeed applications. These findings show the possibility of incorporating fungal fermentation into circular biorefinery strategies, enabling the valorization of food wastewaters while producing sustainable alternative protein sources for aquaculture. However, additional research may be required to more accurately assess the safety and quality of fungus biomass as a feed ingredient, including the monitoring of mycotoxins.

## Data Availability

The datasets presented in this study can be found in online repositories. The names of the repository/repositories and accession number(s) can be found in the article/Supplementary material.
