# Supplementary material for: Bioconversion of food wastewater into Penicillium roqueforti AF26 biomass proteins suitable for aquafeed
Source: Front Microbiol. 2026 Jul 29;17:1857110. doi: 10.3389/fmicb.2026.1857110 (PMC13462464; doi:10.3389/fmicb.2026.1857110)
Supplement: Supplementary file 1 [file Data_Sheet_1.DOCX]

SUPPLEMENTARY MATERIAL S3 Partial β-tubulin gene sequence obtained from the isolate AF26.

CNTTTTTTCGCGTTGGGTACCAATTGACAAATTACTAACTACATTACAGGCNAACCATTTCTGGAGAGCACGGTCTCGATGGCGATGGACAGTAAGTCTAAAGATGATGGGCTTTCCGGTGGTTCACATGTCTAATATCTTGCTAGGTACAATGGAACCTCCGACCTCCNGCTCGAGCGTATGAACGTCTACNTCAACCATGTGAGTACCGCGACTGGGCATTGATTAAGCGTGCACCATCTGATCGGATTTTTTTTTCTTAATCTAGGCCAGCGGTGACAAGTACGTTCCCCGTGCCGTTCTGGTCGATTTGGAGCCCGGTACCATGGACNCCNTCCGCTCTGGTCCTTTCNNCAAGCTTTTCCNCCCCNANNNCTTNNTCTTCNNTCANTCTGGTGCTNNNNACAACTGGNCCAANGGT
