# Supplementary material for: Bioconversion of food wastewater into Penicillium roqueforti AF26 biomass proteins suitable for aquafeed
Source: Front Microbiol. 2026 Jul 29;17:1857110. doi: 10.3389/fmicb.2026.1857110 (PMC13462464; doi:10.3389/fmicb.2026.1857110)
Supplement: Supplementary file 3 [file Table_2.docx]

SUPPLEMENTARY MATERIAL S2 Factors and their levels for a 2^2^ full factorial design, using an organic nitrogen source.

| Factor | Level (-) | Level (+) |
| --- | --- | --- |
| A (Wastewater) | Citrus wastewater | Yogurt whey |
| B (Nitrogen source) | − | CO(NH₂)₂ |
